# Treatment evaluation to improve preventing mother to child transmission among women with syphilis

**DOI:** 10.1038/s41598-019-56095-6

**Published:** 2019-12-20

**Authors:** Tian Gong, Yan Shao, Juning Liu, Qianlan Wu, Rong Xu, Li Sun, Xiaoju Peng

**Affiliations:** Suzhou Maternal and Child Healthcare Center, Suzhou Municipal Hospital, the Affiliated Suzhou Hospital of Nanjing Medical University, Suzhou, China

**Keywords:** Bacterial infection, Health policy

## Abstract

The aim of this study was to evaluate the effectiveness of preventing mother to child syphilis transmission to improve pregnancy outcomes. We performed a retrospective analysis of municipal databases of mother-to-child syphilis transmission. Pregnant women with syphilis were included. Group specific pregnancy outcomes were analyzed according to treatment. A total of 28 pregnant women were diagnosed with syphilis in 2012; 321 were diagnosed with syphilis in 2018. A prevalence of 0.14% was observed amongst pregnant women in Suzhou city from 2012–2018. Primary treatments included benzathine penicillin, ceftriaxone sodium or erythromycin when patients were allergic to Benzathine penicillin. The treatment coverage was 81.57%, and only 52.86% of pregnant women were adequately treated. Adverse pregnant outcomes were higher amongst untreated women. Expanding early screening coverage and promoting treatment were key to improving pregnancy outcomes amongst women with syphilis.

## Introduction

Syphilis is a systemic, sexually transmitted disease caused by the bacterium *Treponema pallidum* that can be transmitted via sexual exposure or from infected mothers to children. Most maternal syphilis infections are latent, and untreated infections can lead to adverse pregnancy outcomes^1,2^. Syphilis remains a public health problem. There were an estimated 988,000 pregnant women infected with syphilis of which 52% experienced adverse pregnancy outcomes in 2016, including spontaneous abortions, stillbirths, preterm or low weight births, and clinical disease in infants^3,4^. Early diagnosis and immediate treatment with penicillin during antenatal care are highly effective in to the prevention of mother-to-child-transmission (PMTCT)^5–7^. The World Health Organization (WHO) launched the global initiative for the elimination of MTCT of syphilis and developed global guidance to reduce congenital syphilis incidence to ≤50 or fewer per 100,000 live births^3^. This included population-level antenatal care coverage for women ≥95%; coverage of syphilis testing of pregnant women ≥95%; and treatment coverage of syphilis-seropositive pregnant women ≥95%^8^. The implementation of PMTCT aims to control and eliminate the MTCT of syphilis. The ultimate goal is to minimize the proportion of untested, untreated or inadequately treated pregnant women to prevent adverse pregnancy outcomes in infants^9^.

Suzhou city is located in central Yangtze River Delta and is famous for its national high-tech industrial base. The population of Suzhou is 10.72 million. 50% of which are migrants. In 2018, the total number of pregnant women and live births in Suzhou were 111488 and 112523 respectively. According to Jiangsu provincial sexual disease surveillance data, adult syphilis increased from 23594 in 2015 to 27256 in 2018, with the incidence of congenital syphilis showing an upward trend during this period. The incidence of syphilis amongst pregnant women has significantly increased with the lack of diagnosis leading to underreported maternal and congenital syphilis cases^10^. The national program of integrated PMTCT of syphilis, HIV and hepatitis B were initiated in Suzhou city from 2011. A clearer understanding of the epidemiology of maternal syphilis and the effects of treatment were necessary for policy decisions and interventional guidance. This study was the first to evaluate the prevalence of maternal syphilis, the effectiveness of treatment, and syphilis-associated pregnancy outcomes in Suzhou city.

## Results

From 2012 to 2018, a total of 1247 pregnant women diagnosed with syphilis in Suzhou according to the PMTCT system were assessed. The increase in annually diagnosed syphilis cases is shown in Table 1. The incidence of pregnant women with syphilis infections increased (*χ*^2^ = 873176, *p* < 0.001). A total of 368 (29.51%) infected pregnant women were local residents, and 879 (70.49%) were migrants. Amongst the 10 reported districts, the incidence was highest in Kunshan city and lowest in the Suzhou industrial park. The number of hospitalized live births decreased from 120,343 to 116,399, but the number of pregnant women diagnosed with syphilis increased from 28 to 321 from 2012 to 2018. Approximately 86.45% of pregnant women were diagnosed during prenatal examinations. In total, 12.27% were diagnosed during delivery. The demographic information of the pregnant women with syphilis infection was showed in Table 2.

**Table 1:** Numbers of live births and syphilis infected women from 2012 to 2018.

Year	Number of live births	Number of syphilis-infected pregnant women	Diagnose time			Number of congenital syphilis
			prenatal examination	delivery	postpartum	
2012	120343	28	23	5	0	0
2013	112947	98	85	12	1	2
2014	135181	155	135	20	0	2
2015	113714	203	174	27	2	1
2016	144437	206	172	29	5	2
2017	130155	236	207	24	5	1
2018	116399	321	282	36	3	0
Total number	873176	1247	1078	153	16	8

**Table 2:** Demographic information of the 1247 pregnant women with syphilis infection.

		Number of infected pregnant women
Age	≤20 years	35
	20–35 years	1051
	35–48 years	161
Education background	primary school and below	88
	junior school	538
	junior high school	332
	bachelor degree and above	238
	no detailed information	51
Transmission route	MTCT	1
	needle stabbing	1
	Occupational exposure	1
	history of drug use	1
	sexual transmission	508
	blood transmission	4
	no detailed information	731
Stage	latent stage	1052
	primary stage	25
	secondary stage	7
	tertiary stage	1
	no detailed information	162
Whether previously infected or not	yes	489
	no	758
Infection status of sexual partner	syphilis infected	181
	not infected with syphilis	532
	not tested	248
	no detailed information	286
Total number of pregnant women with syphilis infection		1247

**Figure 1:**
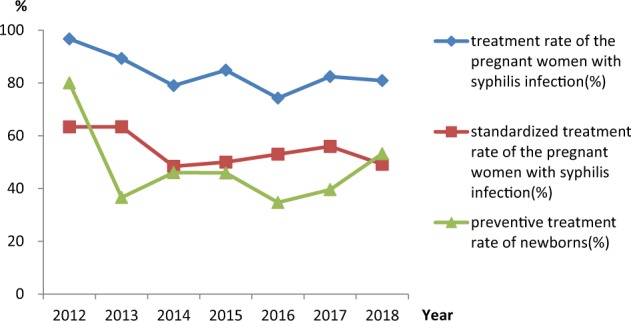
Treatment rates of pregnant women and newborns. (*p* ≤ 0.05 was considered statistically significant.

The number of puerpera with syphilis infections and live births increased from 2012 to 2018. The treatment of pregnant women with syphilis decreased (*χ2* = 18.29, *p* = 0.006), but standardized treatment rates remained unchanged (*χ2* = 10.22, *p* = 0.116), Table 3, Fig. 1.

**Table 3:** Treatment rates of pregnant women and their newborns.

Year	Number of puerpera with syphilis infection	Number of live births	Number of puerperae with treatment (n,%)	Number of puerperae with standardized treatment (n,%)	Number of newborns needed to be treated	Number of newborns with preventive Treatment (n,%)
2012	30	29	29 (96.67)	19 (63.33)	10	8 (80.00)
2013	112	112	100 (89.29)	71 (63.39)	41	15 (36.59)
2014	157	152	124 (78.98)	76 (48.41)	76	35 (46.05)
2015	178	174	151 (84.83)	89 (50.00)	85	39 (45.88)
2016	202	207	150 (74.26)	107 (52.97)	98	34 (34.69)
2017	193	196	159 (82.38)	108 (55.96)	86	34 (39.53)
2018	246	247	199 (80.89)	121 (49.19)	126	67 (53.17)

A total of 106 spontaneous abortions occurred, of which 11 died within 7 days of birth and 17 stillbirths occurred. Adverse pregnancy outcomes were higher amongst untreated women (*χ2* = 213.96, *p *< 0.001). Detailed information is shown in Table 4.

**Table 4:** Comparison of pregnancy outcomes according to treatment groups.

Treatment group	Abortion (n,%)	Live births (n,%)	Died within seven days after birth (n,%)	Stillbirth (n,%)	Total
Untreated	78 (73.59)	193 (17.11)	5 (45.45)	10 (58.82)	286
Treated	27 (25.47)	323 (28.63)	5 (45.45)	3 (17.65)	358
Standardized treatment	1 (0.94)	612 (54.26)	1 (9.10)	4 (23.53)	618
Total	106	1128	11	17	1262

(*p* ≤ 0.05 was considered statistically significant).

## Discussion

According to the National Statutory Infectious diseases reports, the prevalence of adult syphilis and maternal syphilis infections have increased in China since 2011. The prevention of syphilis transmission from mother to children is therefore of high priority. In this study, the average prevalence of syphilis in pregnant women was 0.14%, which was similar to the reported prevalence of Europe (0.16%)^11^. The rate of maternal syphilis in Suzhou was 0.28% in 2018, which was higher than China (0.24%)^12^ and the Mediterranean (0.06%), but lower than Latin America (1.1%) and India (0.57–0.78%)^11,13,14^. The increased prevalence of maternal syphilis was partly due to the accumulation of the previously infected women and the inflow of the migrant pregnant women with syphilis infections. According to the requirement of PMTCT of syphilis program, free syphilis screening should be provided to all pregnant women during initial prenatal care examinations, but full coverage was not achieved. Based on our studies, most syphilis cases in infected pregnant women occur in migrants and the unemployed, in which educational background are junior school or below. These women do not value prenatal care and lack access to effective treatment. In addition, women with syphilis infections suffer from the stigma and sociological discrimination that occur for other sexually transmitted diseases (STDs). This physiological status restricts the awareness of pregnant women seeking professional medical healthcare.

Vertical transmission is dependent on the stages of maternal infection, for which the risk is highest during the primary and secondary stages of infection, followed by asymptomatic syphilis during pregnancy^1,15^. Adverse outcomes in pregnant women with syphilis infections are common, particularly for those who are inadequately treated^16–18^. A single dose of penicillin is the first-line treatment and most cost-effective treatment for women with syphilis during pregnancy^19,20^. Since the launch of the global initiative of eliminating the MTCT of syphilis, the Chinese government has integrated the PMTCT of HIV and syphilis with maternal healthcare management. The screening and surveillance of prenatal syphilis has improved in Suzhou city. From 2012 to 2018, the number of infants diagnosed with congenital syphilis has decreased. However, due to deliberate concealment and avoidance of syphilis detection, delayed diagnosis leads to delayed treatment. Consistent with previous studies^21–23^, to improve patient compliance, a efforts to promote the importance of pregnant syphilis screening and the benefits of standardized treatment have been employed. The syphilis screening rates at the first trimester have improved from 82.14% to 87.85% during the past 7 years in Suzhou. However, treatment rates of 16.32% were recorded in 2018 which were lower than those of 2012. The adverse pregnancy outcomes amongst women lacking treatment were higher than those treated with standard care.

Despite the prevalence of syphilis in Suzhou city being lower than high-epidemic cities in China, the Jiangsu province was highly afflicted due to the large migrant population. Migrants are attracted by economic development. These relationships should be assessed in more detail in future studies.

This was the first study investigating the prevalence of syphilis and its characteristics amongst pregnant women in Suzhou, There were several limitations. Firstly, we could not compare pregnancy outcomes between healthy women and women with syphilis due to the lack of data availability. Secondly, the influence of a partner’s infection status on infant health was not assessed due to limited information. Finally, only selected adverse pregnancy outcomes were recorded in the PMTCT system, meaning the effectiveness of PMTCT was not fully evaluated.

In summary, we demonstrate that full-course and adequate treatment improves pregnancy outcomes and infant health. The priority of PMTCT of syphilis is to improve early screening coverage and the compliance of pregnant women with medication in Suzhou city, particularly in the migrant population.

## Methods

### Data collection

Data were extracted from the municipal PMTCT system from 2012 to 2018. According to the requirements of the PMTCT program, syphilis counseling and testing were combined with routine prenatal healthcare. All pregnant women underwent syphilis counseling and testing upon their initial prenatal healthcare appointment. Positive results in syphilis rapid plasma regain (RPR)/toluidine red unheated serum test (TRUST) and treponemal pallidum particle agglutination (TPPA)/enzyme linked immunosorbent assay (ELISA) tests led to a diagnosis of maternal syphilis infection. Syphilis infected women were provided free penicillin in the first and the third trimester, respectively. All pregnant women were screened prior to delivery. For children born to syphilis infected women, syphilis screening and preventive treatment were performed if the mothers failed to receive two courses of full-time penicillin during the first and third trimester of pregnancy, or if their mothers received full-time penicillin treatment during pregnancy. Newborns maintained reactive TRUST or RPR serum titers that were 4-times lower than the mothers prior to delivery. Exposed children were followed up every three months until a syphilis diagnosis was excluded. Information on infected pregnant women, pregnancy outcomes and follow ups of exposed children were recorded in the PMTCT system.

### Statistical analysis

The database was exported in Excel and analyzed using SPSS 17.0 software (Chicago Illinois, USA). Demographic characteristics of syphilis infected women were described. Categorical variables are presented as numbers and frequencies. Statistically significant differences between the groups were evaluated using the *χ*^2^-test or *Fisher’s* exact test as appropriate. *P* < 0.05 was considered statistically significant.

### Ethical considerations

This study was approved by the Ethics committee of Suzhou Municipal Hospital. All infected mothers were required to complete a routine questionnaire upon receiving syphilis screening and testing at their first antenatal care or delivery. Informed consent was obtained from syphilis infected mothers. Prenatal syphilis screening and testing processes followed the guidelines and regulations of the integrated National Preventing Mother to Child Transmission Program of HIV, Syphilis and Hepatitis B. In the final database, only mothers and infant numbers were listed. All personal information was kept confidential.

## Data Availability

The datasets generated and analyzed during the current study are available from the corresponding author on reasonable request.
